# High-Efficiency Broadband Planar Array Antenna with Suspended Microstrip Slab for X-Band SAR Onboard Small Satellites

**DOI:** 10.3390/s22010252

**Published:** 2021-12-30

**Authors:** Kyei Anim, Patrick Danuor, Seong-Ook Park, Young-Bae Jung

**Affiliations:** 1 Electrical and Computer Engineering Department, Drexel University, Philadelphia, PA 19104, USA; ak4259@drexel.edu; 2 Electronics Engineering Department, Hanbat National University, Daejeon 34158, Korea; 30211162@edu.hanbat.ac.kr; 3 Electrical Engineering, Korea Advanced Institute of Science and Technology, Daejeon 34141, Korea

**Keywords:** asymmetric corporate feeding, broadband, high efficiency, high gain, parasitic patch, sidelobe, suspended microstrip slab, synthetic aperture radar

## Abstract

In this paper, a high efficiency broadband planar array antenna is developed at X-band for synthetic aperture radar (SAR) on small satellites. The antenna is based on a multi-layer element structure consisting of two dielectric substrates made of Taconic TLY-5 and three copper layers (i.e., the parasitic patch (top layer), the active patch (middle layer), and the ground plane (bottom layer)). The parasitic patch resides on the bottom surface of the upper TLY-5 substrate while the active patch is printed on the top surface of the lower substrate. A Rohacell foam material is sandwiched between the top layer and the middle layer to separate the two dielectric substrates in order to achieve high directivity, wideband, and to keep the antenna weight to a minimum as required by the SAR satellite application. To satisfy the required size of the antenna panel for the small SAR satellite, an asymmetric corporate feeding network (CFN) is designed to feed a 12 × 16 planar array antenna. However, it was determined that the first corporate feed junction at the center of the CFN, where higher amplitudes of the input signal are located, contributes significantly to the leaky wave emission, which degrades the radiation efficiency and increases the sidelobe level. Thus, a suspended microstrip slab, which is simply a wide and long microstrip line, is designed and positioned on the top layer directly above that feed junction to prevent the leaky waves from radiating. The experimental results of the antenna show good agreement with the simulated ones, achieving an impedance bandwidth of 12.4% from 9.01 to 10.20 GHz and a high gain above 28 dBi. The antenna efficiency estimated from the gain and directivity eclipses 51.34%.

## 1. Introduction

Synthetic aperture radar (SAR) satellites provide a wealth of information on sea and land surfaces [1]. The interest in the development of these systems has seen an upsurge in recent years due to the advantages such as day and night, and all-weather operation, and the suitability to various platforms, such as a missile, satellite, and pilotless aircraft [1,2,3,4]. Nonetheless, the most important component in determining the overall system performance of the space-based SAR systems is the antenna, and thus the SAR system requirements impose great demands on the antenna performance.

In general, the design concepts researched for SAR antenna panels on small satellites are high efficiency, high gain, broadband operation [3], low sidelobe levels [5], and low weight. Thus, various types of antennas such as Yagi-Uda, slotted-waveguide, and microstrip antennas have been investigated for the SAR application [6]. Due to their advantages such as their compact size, lightweight, low profile, low cost, ease of manufacturing, and superior ability to integrate with front-end RF circuits, microstrip patch antennas (MPAs) [5,7,8,9] are widely developed for modern civilian SAR systems. However, the MPAs are inherently narrowband, having a relatively low gain and efficiency. For this reason, various techniques have been investigated and reported in [1] in terms of the radiating element to achieve high-performance SAR antennas concerning bandwidth, efficiency, and gain. In terms of the feeding structure, several examples of corporate or parallel feeds [10,11,12,13] are employed in the microstrip arrays as excitation networks due to the advantages of broadband operations, design flexibility, and ease of vertical integration to form a two-dimensional array [14]. The corporate feeding network (CFN) is simply a combination of the multiport microstrip junctions (often referred to as power dividers) such as the T- and cross-junction power dividers. These microstrip junctions are connected with uniform microstrip lines, which are theoretically assumed to be loss-free transmission lines [15]. However, the microstrip arrays with CFNs often experience low radiation efficiency and high sidelobe levels (SLLs). This can be attributed to the strong leaky-wave and surface-wave emissions resulting from the microstrip junctions of the CFN and, to some degree, from the dielectric substrates that severely affect the SAR system performance due to degraded efficiency to risk mission failure.

In this paper, a high-performance microstrip array antenna is designed at the X-band by implementing variants of design techniques to achieve the desirable bandwidth, efficiency, gain, and weight for the SAR satellite. Thus, the proposed antenna is based on a multi-layer element structure that has two TLY-5 dielectric substrates and two patches functioning as the active and parasitic radiators (which are separated by a relatively thick Rohacell foam material). This configuration helps to achieve a high gain and efficient antenna with broadband operations which is lightweight and well suited for the small SAR satellites. The Rohacell material has a dielectric constant close to air and a very low tangent loss in order to be as lightweight as possible and to maintain a high efficiency. Ultimately, a full-corporate fed 12 × 16 planar array antenna was developed with the adoption of suspended microstrip slab structure to mitigate the impact of the leaky waves due to the higher amplitudes of the input signal on the T-junction power dividers. The antenna, therefore, operates in a band of 9.01−10.20 GHz and achieves a peak gain above 28 dBi with an estimated efficiency of about 51.34%. The proposed antenna can be used in SAR technology applications for ground sensing and monitoring.

## 2. Proposed Array Antenna Configuration

### 2.1. Antenna Geometry

**Figure 1:**
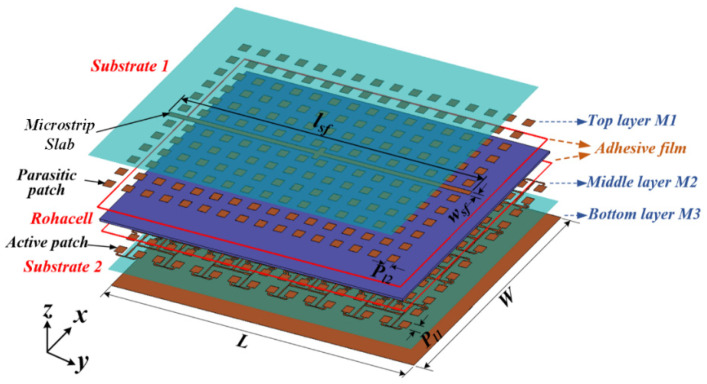
Exploded 3D model of the proposed array antenna. (P_l1_ = 10.23 mm, P_l2_ = 10.4 mm, l_sf_ = 371 mm, w_sf_ = 6 mm, L = 371 mm and W = 276 mm).

The geometry of the proposed array antenna shown in Figure 1 was developed based on the following antenna specifications for small-satellite SAR [16].

- Antenna Size: (Az × El) 371 mm × 276 mm
- Gain ≥ 28.5 dBi
- Frequency: 9.65 GHz [BW: 300 MHz]
- Efficiency > 50%
- Reflection Coefficient ≤ −15 dB
- Beamwidth: Az > 4.0°, El > 5.8°
- Sidelobe Level: Az < −13 dB, El < −13 dB
- Polarization: linear (Vertical)
- Port: SMA (F)
- Antenna Panel Launch Mass: 0.53 kg

The antenna is a multilayered 12 × 16 array structure built on two dielectric substrates made of Taconic TLY-5 (i.e., substrate 1 and substrate 2, ɛ_r_ = 2.2, tan δ = 0.0009) with a relatively thick Rohacell material (ɛ_r_ = 1.07, tan δ = 0.0017) sandwiched between them. The thickness of the two substrates is given as h_sub1_ = h_sub2_ = 0.508 mm. The antenna contains three copper layers (i.e., a top layer M1, middle layer M2, and bottom layer M3). In the structural design, the middle layer M2 constitutes the corporate-fed square-shaped microstrip patches that are employed due to their simple structure and generally good performance, and they reside on the top surface of the lower substrate (as shown in Figure 1). These are the active patches that couple power electromagnetically through the Rohacell material to the top layer, M1, consisting of parasitic square-shaped patches and a suspended microstrip slab structure at the backside of the upper substrate (i.e., substrate 1). The copper cladding at the bottom layer, M3, serves as the ground plane.

From the configuration of the proposed array antenna, as shown in Figure 1, it can be seen that adhesive films are used as a bonding agent to glue the two TLY-5 dielectric substrates to the Rohacell material. With this configuration, the array antenna achieves high gain, high efficiency, and broadband performances more than many existing planar array antennas for SAR applications with stack-up topology. Meanwhile, the weight of the antenna is kept to the minimum owing to the lightweight property of the Rohacell foam layer. A suspended microstrip slab structure is employed in the design to suppress any parasitic radiations attributable to leaky waves from the central most power divider where most of the high-amplitude signals are concentrated.

### 2.2. Antenna Element Design

**Figure 2:**
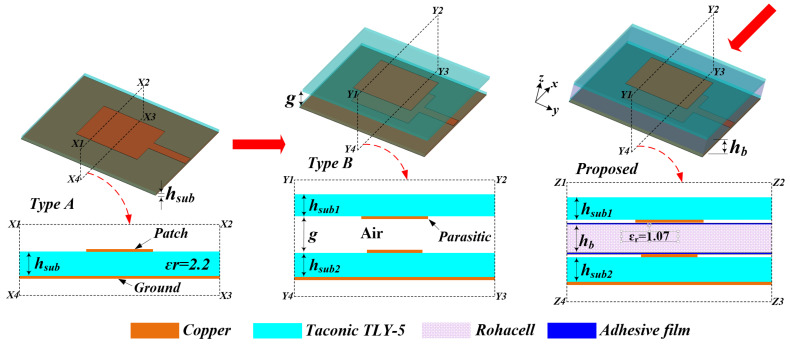
Three different types of radiating elements. (h_sub1_ = 0.508 mm, h_sub2_ = 0.508 mm, h_b_ = 2.5 mm, H_air_ = 2.536 mm, and adhesive film thickness = 0.2 mm).

**Figure 3:**
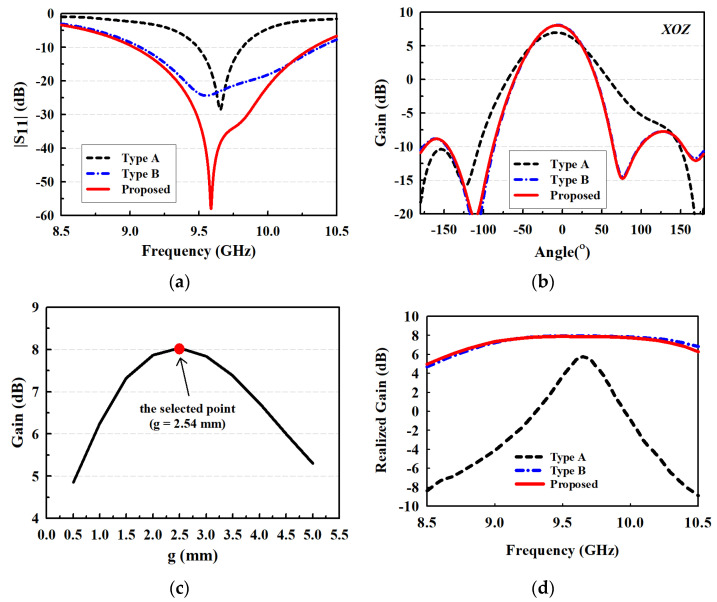
Simulation results of three different radiating elements showing (**a**) |S_11_|, (**b**) gains at 9.65 GHz, (**c**) gain versus the air-gap width between the two substrates at 9.65 GHz and (**d**) realized gain variation.

The three different types of radiating elements in Figure 2 depict the evolution of the proposed case, which starts with a Type A microstrip antenna consisting of a square-shaped patch that is directly fed by a feeding line printed on a dielectric substrate with a backed ground plane. According to the simulation results in Figure 3a, a relatively narrow bandwidth (i.e., around 300 MHz at 9.65 GHz) is achieved for the −10-dB |S_11_| frequency band. In Figure 3b, the gain of this conventional antenna-Type A is about 6 dBi. However, the intrinsic resonant behavior of Type A does not necessarily satisfy the antenna requirements for a high-resolution SAR based on the geometrical and the slant-range resolution (*P_r_*) analysis of the overall antenna system using Equation (1) [17]:

(1) $$\rho_{r}=\frac{c}{2B}$$

where *c* denotes the speed of light and *B* is the system bandwidth. Thus, it implies that a SAR system, which requires a resolution better than 0.2 m, should have a bandwidth of more than 1 GHz.

To increase the impedance bandwidth and directivity of antenna-Type A, a parasitic patch, which is mounted on the bottom side of a superstrate layer and has a resonance frequency close to the resonant frequency of Type A, is placed close to Type A to produce the antenna-Type B (as shown in Figure 2). Thus, the antenna-Type B becomes a parasitically coupled stack configuration with an air gap between the two patches to result in a much better bandwidth than Type A (as shown in Figure 3a). In addition, a higher gain can be observed in the frequency band of 9.1–10.4 GHz (as shown in Figure 3b, Type B). Thus, by the adjusting of the air-gap width, *g,* between the two patches, the parasitic patch appears to direct the radiation from the active patch toward the boresight direction to maximize the directivity of the antenna (as reported in Figure 3c). The curve of the gain around 8 dBi is obtained when the value of g is around 2.54 mm. However, since it is impractical to suspend the superstrate layer of Type B in air, the simplest approach to the design is to sandwich a Rohacell structural foam between the two substrates with adhesive films bonding them together to achieve the proposed radiating element in Figure 2. The Rohacell material was selected due to its low tangent loss of 0.0017 and lightweight property due to the fact that its dielectric constant is similar to that of air. As a result, the proposed radiating element exhibits a bandwidth performance similar to that of Type B, as shown in Figure 3a,b (i.e., −10 dB |S_11_| bandwidth of 1.3 GHz). Again, from Figure 3d, it can be observed that both Type B and the proposed element antenna have a similar gain performance. Moreover, both Type B and the proposed antenna have a broader gain bandwidth compared to the Type A.

### 2.3. Array Design

**Figure 4:**
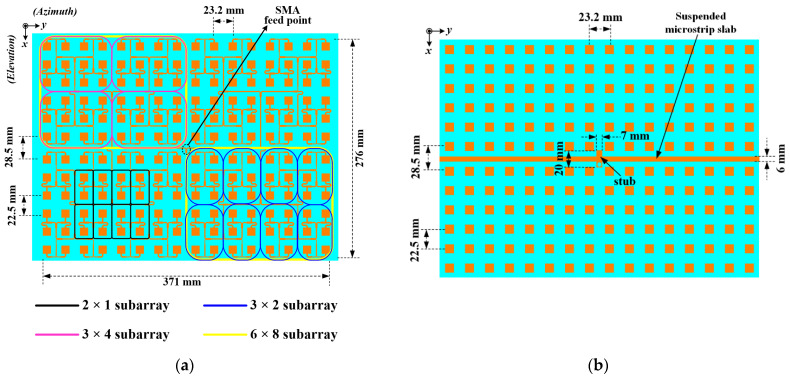
Configuration of the developed 12 × 16 array antenna showing (**a**) the middle layer M2 printed on the lower substrate and (**b**) the top layer M1 residing on the bottom side of the upper substrate.

In order to satisfy the required size of 371 mm × 276 mm of the antenna specifications of SAR, a corporate-fed 12 × 16 planar array was developed based on the proposed radiating element. Figure 4 shows the configuration of the proposed antenna.

As seen in Figure 4a, the antenna has two rudimentary subarrays 1 × 2 and 2 × 1 to construct several 3 × 2 subarrays. The 3 × 2 subarrays are further utilized to construct 3 × 4 subarrays, which were subsequently adopted for developing 6 × 8 subarrays. Altogether, four 6 × 8 subarrays are positioned in a sequential rotation scheme to achieve a rectangular-shaped radiating aperture to contain the full 12 × 16 array with the feed point of the feed network at the center.

The active patches, which are printed on the top surface of the lower TLY-5 substrate, have an inter-element spacing of 0.59λ_0_. Thus, the centre-to-centre distance between adjacent radiating elements is 18.34 mm in the *XOZ*-plane (Elevation plane) where λ_0_ denotes the wavelength corresponding to the centre operating frequency. However, the two middle rows are 0.87λ_0_ = 27.05 mm apart to make room for the feeding network. In the *YOZ*-plane (Azimuth plane), the patches assume a centre-to-centre spacing of 0.62λ_0_ = 19.27 mm, as shown in Figure 4a.

Similarly, the elemental spacing between the parasitic patches at the backside of the upper substrate is given as 18.34 mm in the *XOZ*-plane, as shown in Figure 4b. Meanwhile, they are separated by a distance of 19.27 mm in the *YOZ*-plane. The spacing between the two middle rows of parasitic patches in the *XOZ*-plane is given as 27.05 mm to align them with their corresponding active patches.

**Figure 5:**
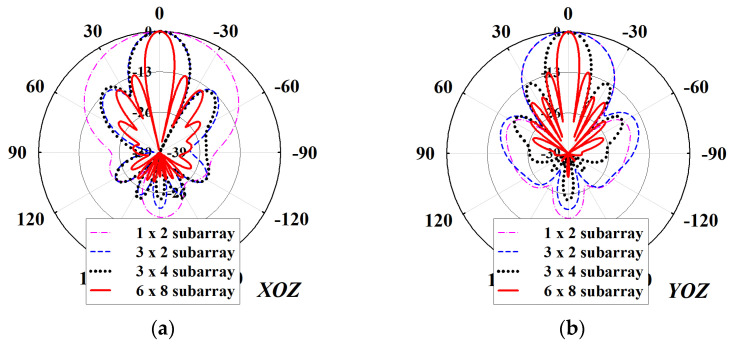
Simulated radiation patterns at 9.65 GHz for all of the subarrays of the proposed antenna: (**a**) *XOZ*-plane, and (**b**) *YOZ*-plane.

To ensure that the proposed array antenna achieves the required sidelobe levels (SLLs) below −13 dB, the individual radiating elements should give out an equal amount of input power. Thus, the antenna is a uniform array having identical active and parasitic patches with a resonant length of P_l1_ = 10.23 mm and P_l2_ = 10.4 mm, respectively, without aperture tapering. Referring to Figure 5, the sidelobe amplitudes increases considerably as the size of the array increases toward the maximum array size in both principal planes. Thus, the full antenna array produces SLLs more than the required −13 dB threshold. This phenomenon is largely attributed to the leaky wave emissions from the power splitters of the CFN particularly the ones closest to the feed point where higher amplitudes of the input signal are located.

### 2.4. Feeding Network

**Figure 6:**
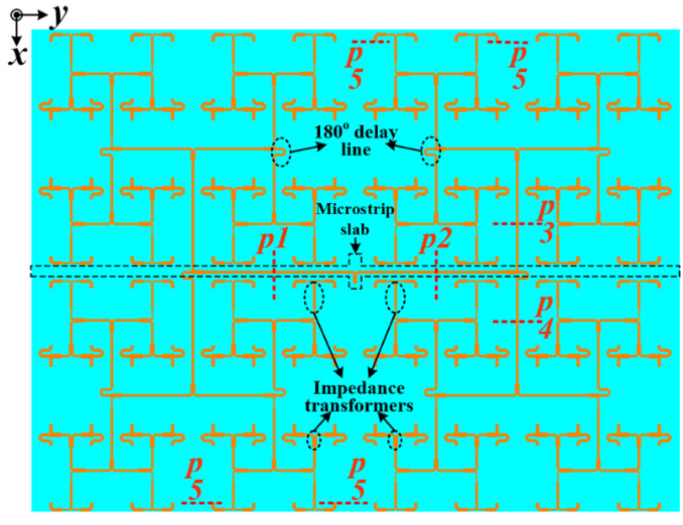
Configuration of the developed feeding network.

In order to overcome the sidelobe problem of the full array antenna, an asymmetric corporate feeding network with a suspended microstrip slab is designed here, as shown in Figure 6.

As can be seen in Figure 6, the corporate feed network is realized by the combinations of both the equal and unequal T-junction power dividers, 180° delay lines, quarter-wavelength transformers, and simple microstrip lines. The corporate feed structure is designed to produce excitations of equal magnitude to all of the elements of the array. The 180° delay lines are employed to excite adjacent patches in the elevation plane, which are geometrically 180° out-of-phase with equal amplitudes but with a 180° phase difference to cancel out the geometric phase difference and ensure that the beams combine constructively. The developed feeding array is fed at its midpoint using a coaxial probe to match the input impedance at 50 Ω. It should be mentioned that a hole is drilled through the substrates of the antenna to make the soldering of the SMA connector possible. In effect, the corporate feeding structure also contributes to the broad banding of the antennas’ operating bandwidth, as expected.

**Figure 7:**
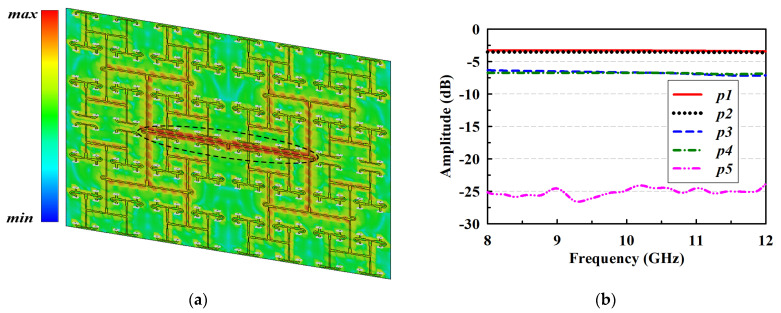
Simulated performance of the developed feeding network; (**a**) current distribution and (**b**) output amplitudes.

Figure 7a plots the simulated current distribution of the feeding network. It shows that higher amplitudes of the input signal are mostly located around the first T-junction power divider at the center of the feed network. Thus, the output amplitudes of P1 and P2 of that T-junction shown in Figure 7b are about −3.5 dB, which means that a loss of 0.5 dB was incurred. This loss is largely due to the leaky wave excitation by the T-junction since the transmission line loss at this point of the network is negligible. It could, therefore, be conjectured that the leaky wave by the first T-junction power divider contributes more to the parasitic radiations that tend to significantly increase the sidelobe level of the antenna than the other T-junction power dividers. The output amplitudes for *P3–P5* are then approximately −6.6, −6.7, and −25 dB, respectively, as shown in Figure 7b.

To alleviate the effect of the leaky wave emission, it is imperative to suppress the parasitic radiations from the T-junction power divider, especially the one closest to the feed point. Thus, a suspended microstrip slab structure (see Figure 4b and Figure 6) is designed and incorporated into the antenna geometry. It is located at the backside of the upper TLY-5 substrate (i.e., the first layer M1) but directly above the most central region of the feeding network where higher amplitude signals are concentrated. With the integration of the suspended microstrip slab, the leaky waves from the first power divider of the feed network parasitically coupled to the microstrip slab structure without radiating.

**Figure 8:**
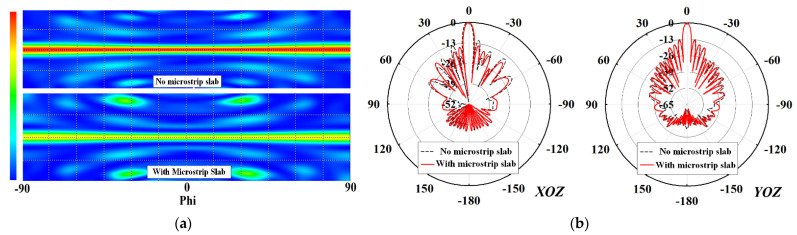
(**a**) Simulated radiation amplitude of the feeding network at 9.65 GHz and (**b**) 2D simulated radiation patterns of the proposed 12 × 16 array antenna with and without the suspended microstrip slab structure at 9.65 GHz.

To validate the practicality of this design technique to decrease the SLLs, a simulated 2D radiation amplitude of the feeding network was plotted in Figure 8a. It can be noticed that the radiation amplitude of the feeding structure is reduced substantially when the microstrip slab structure was implemented. Thus, the SLLs are decreased substantially from −12.4 dB to −15 dB, especially in the *XOZ*-plane (as depicted in Figure 8b) to satisfy SAR antenna requirements.

## 3. Results and Discussion

**Figure 9:**
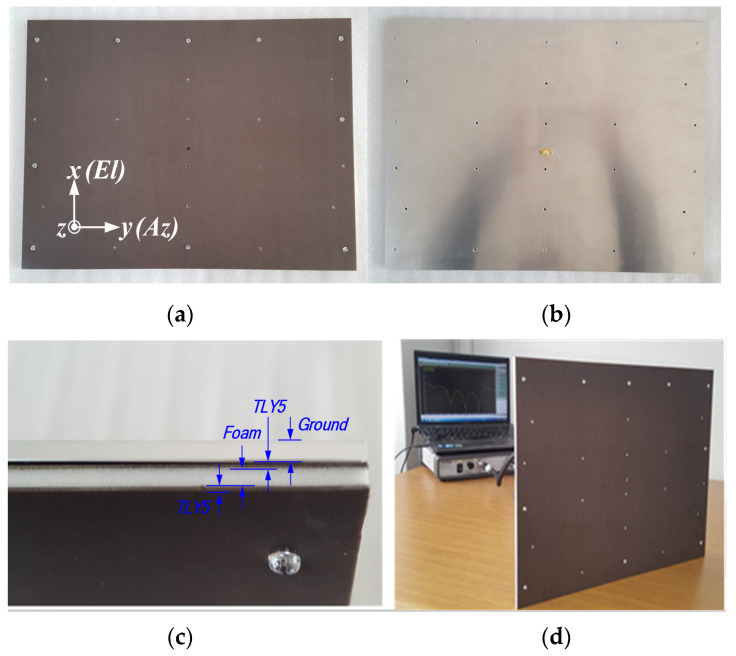
Photograph of the fabricated array antenna: (**a**) front view; (**b**) back side; (**c**) side view; (**d**) measurement setup in the lab.

**Figure 10:**
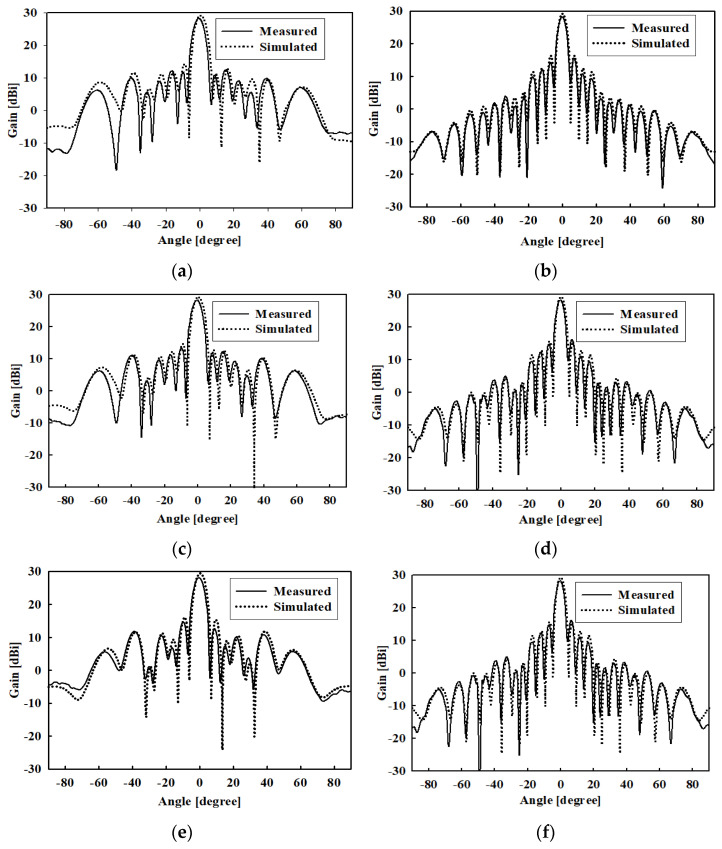
Measured and simulated farfield radiation patterns of the proposed array antenna. (**a**) 9.3 GHz, *XOZ*-plane, (**b**) 9.3 GHz, *YOZ*-plane, (**c**) 9.65 GHz, *XOZ*-plane, (**d**) 9.65 GHz, *YOZ*-plane (**e**) 9.9 GHz, XOZ-plane, and (**f**) 9.9 GHz, *YOZ*-plane.

Figure 9 shows the photograph of the fabricated prototype of the proposed 12 × 16 planar array antenna. The measured and simulated radiation patterns at 9.3, 9.65, and 9.9 GHz in both *XOZ*- and *YOZ*-plane are shown in Figure 10a–g, while the measured *XOZ*-plane (El) half power beamwidths (HPBWs) are 6.0°, 5.8°, and 5.7° at these frequency points, respectively. The measured YOZ-plane (Az) HPBWs are 4.3°, 4.2°, and 4.1° at the three frequency points, respectively. The experimental and predicted outcomes agree well. The sidelobe levels (SLLs) are below −13 dB at the three frequency points expected of uniform arrays. The total antenna efficiency was estimated to be above 51.34% from the gain and directivity of the array antenna.

**Figure 11:**
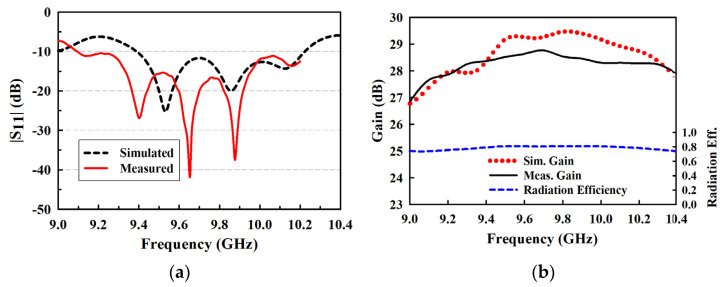
Experimental results of the proposed 12 × 16 planar array antenna. (**a**) S_11;_ (**b**) gain and efficiency.

The experimental results of |S_11_| plotted in Figure 11a shows a measured impedance bandwidth of 1.19 GHz (i.e., 12.39%, 9.01−10.20 GHz, |S_11_| < −10 dB), which ironically outperform the simulated one (8.47%, 9.39−10.22 GHz). The measured peak gain is 28.67 dBi at 9.65 GHz, as shown in Figure 11b. The disparity between the measured and simulated gain is largely due to the minor fabrication inaccuracy and measurement errors. It should be noted that there is a good correlation between the simulated and measured impedance bandwidths. However, the shift in resonant peak points could be resulting from the connector losses during measurement, the misalignment of the layers in the antenna structure, and possibly the presence of air gaps between layers. The antenna has a radiation efficiency above 80% in the band of 9.5−10 GHz (the band of interest).

Table 1 shows a comparison between the proposed antenna array and leading-edge planar SAR antennas. Among all of the designs, the proposed planar array has the simplest geometry and requires no additional assembly during fabrication (as opposed to the other listed multilayered designs). Although the antenna achieves high efficiency that is within the acceptable limits for developing SAR antennas, it is lower than [2,9]. It has gain performance comparable to [2] when the size of the array is taken into account. The proposed antenna exhibits broader impedance bandwidth than most of the antennas in the table except for antennas [9,18]. It can be noted that the overall performance of the proposed antenna is very satisfactory to make it a suitable candidate for high-resolution synthetic aperture radar applications.

## 4. Conclusions

In this paper, a corporate-fed 12 × 16 planar array antenna has been proposed for ground sensing and monitoring applications in SAR technology. The antenna is based on a stack-up element structure with Rohacell material sandwiched between two TLY-5 substrates. The proposed antenna array is suitable for an X-band SAR on a small satellite platform due to its simple structure, high efficiency, broadband, and low weight. The incorporation of the Rohacell structural layer into the antenna design helps to broadband the impedance bandwidth of a traditional MPA. Meanwhile, due to the low tangent loss of the used Rohacell material and dielectric constant close to that of air, the proposed antenna results in both high efficiency and a light weight, respectively. However, owing to the corporate feeding structure of the full array, the parasitic radiations (due to the leaky wave emissions by the power dividers of the feed) tend to increase the sidelobe levels (SLLs) of the antenna. Thus, a suspended microstrip slab structure was implemented to subdue the leaky-wave radiations to decrease the SLLs within the acceptable limits for developing SAR antennas.

## Figures and Tables

**Table 1:** Comparison of proposed antenna with some previously reported X-band SAR array antennas.

Antenna	Frequency Band	Array Size	Antenna Size (mm)	Polarization	Bandwidth	Max. Gain	Antenna Efficiency	Sidelobe Level
[2]	X	24 × 32	678 × 700	Circular	1.3%	34.9 dBi	54%	11 dB
[9]	X and Ku	4 × 6	77 × 79	Horizontal and Vertical	30%, 23% X-band, Ku-band	14.1 dBi, X-band. 12.7 dBi, Ku-band	80%	6 dB
[18]	X	1 × 6	20 × 20	Horizontal and Vertical	16%	8 dBi	-	-
[19]	X and S	S-band 2 × 1 X-band 7 × 4	100 × 140	Horizontal and Vertical	5% in both X- and S-bands	-	-	10 dB
[20]	X	1 × 8	150 × 24	Horizontal and Vertical	7.8%	15 dBi	-	25 dB
[21]	X and K	6 × 6	150 × 150	Horizontal and Vertical	10.36% ~X-band 1.45% ~K-band	24.2 dBi, X-band and 17.4 dBi at K-band	85%	18 dB
[22]	X, Ku and Ka	X-band 2 × 2 Ku-band 4 × 4 Ka-band 4 × 4	-	Horizontal and Vertical	3.6% ~X-band 6.7% ~Ku-band 5.3% ~Ku-band	19.2 dBi	80%	12 dB
[23]	C and K	C-band 2 × 2 X-band 4 × 4	-	Circular		14.5 dBi, C-band and 17.5 dBi, X-band	55%	15 dB
Proposed	X	12 × 16	371 × 276	Vertical	12.39%	28 dBi	51.3%	13 dB

## Data Availability

Not applicable.
